# Impact of capitation prepayment on the medical expenses and health service utilization of patients with coronary heart disease: a community policy intervention program in a county in China

**DOI:** 10.1186/s12889-023-17161-x

**Published:** 2023-11-10

**Authors:** Jincao Yan, Yunke Shi, Jiani Zhang, Siwei Chen, Xinran Huo, Yue Shen, Ning Zhang

**Affiliations:** School of Public Health, Capital Medical University, Beijing, 100069 China

**Keywords:** Capitation prepayment, Coronary heart disease, Propensity score matching (PSM), Difference-in-difference (DID) model, Reform impact, Medical expenses, Health service utilization

## Abstract

**Background:**

Medical costs have been rising rapidly in recent years, and China is controlling medical costs from the perspective of health insurance payments.

**Objectives:**

To explore the impact of the capitation prepayment method on medical expenses and health service utilization of coronary heart disease (CHD) patients, which provides a scientific basis for further improvement of the payment approach.

**Methods:**

The diagnosis records of visits for CHD in the database from 2014 to 2016 (April to December each year) were selected, and two townships were randomly selected as the pilot and control groups. Propensity score matching (PSM) and difference-in-difference (DID) model were used to assess changes in outpatient and inpatient expenses and health service utilization among CHD patients after the implementation of the capitation prepayment policy.

**Results:**

There were eventually 3,900 outpatients and 664 inpatients enrolled in this study after PSM. The DID model showed that in the first year of implementing the reform, total outpatient expenses decreased by CNY 13.953, drug expenses decreased by CNY 11.289, as well as Medicare payments decreased by CNY 8.707 in the pilot group compared to the control group. In the second year of implementing the reform, compared with the control group, the pilot group had a reduction of CNY 3.123 in other expenses, and a reduction of CNY 6.841 in Medicare payments. There was no significant change in inpatient expenses in the pilot group compared to the control group, but there was an increase of 0.829 visits to rural medical institutions, and an increase of 0.750 visits within the county for inpatients.

**Conclusions:**

The capitation prepayment method has been effective in controlling the outpatient expenses of CHD patients, as well as improving the medical service capacity of medical institutions within the Medical Community, and increasing the rate of inside county visits for inpatients.

## Background

As the population ages and the disease spectrum changes, the prevalence of chronic diseases is on the rise. The prevalence of chronic diseases among Chinese residents aged ≥ 15 years was 342.9‰ in 2018 [1], and the burden of medical costs for patients with chronic diseases continues to increase. Coronary heart disease (CHD), or coronary atherosclerotic heart disease, is a chronic non-communicable disease and is one of the main sources of disease burden in Chinese residents [2]. According to the results of the Fifth Health Services Survey in China, the prevalence of CHD in the population aged ≥ 15 years was 10.2‰ nationwide [3]. The mortality of CHD is also increasing year by year, and the mortality of CHD was 135.08/100,000 for urban residents, and 148.19/100,000 for rural residents in China in 2021 [1].

Direct and indirect costs induced by disease contribute to the continuing rise in healthcare expenses. China’s total per capita medical expenditure in 2021 is 1.84 times higher than that in 2015, reaching CNY 5,440. Rising healthcare expenses and inadequate health outcomes are serious challenges worldwide [4]. There are plenty of reasons for the rapid growth of medical expenses, including uncontrollable factors, such as population aging, changes in the disease spectrum, medical technology advancement, price inflation and public health awareness enhancement, the insufficiency of cooperation and competition among institutions within the medical and health system, and duplicate examinations and treatments among institutions. International experience has shown that the effectiveness of payment reform on healthcare cost savings is more durable [5].

The sample of this study–FN County is located in the northwest of Anhui Province, where are 28 townships and a provincial economic development zone, and 328 village (neighborhood) committees, with a household population of 1,733,000 and a resident population of 1,174,000 in 2021. The county is a national poverty-stricken county until 2020, which is also a county with large agricultural population and labor export, scarce medical resources, and high rate of out-of-county visits. In order to control the rapid rise of medical costs, FN County has been the pioneer in China since 2015 to initiate the reform of the County Medical Service Community (“Medical Community”), carry out pilot construction of the Medical Community, and launch the implementation of the capitation prepayment for medical insurance payment. The medical insurance agency implements the operation and management method of “total control, quarterly pre-allocation, year-end accounting, non-overspending principle and retention of surplus” for the medical insurance funds of the Medical Community. The capitation prepayment is a payment method that the health insurance provider pre-packages the health insurance fund to the medical community based on the number of people covered by the medical community [6]. The capitation prepayment method adjusts the competitive relationship between tiered medical institutions within the Medical Community to a cooperative relationship, which transforms the tiered medical institutions from an “organizational community” to a “service community” via a “community of interest” and shifts the focus of service of tiered medical institutions from disease treatment to disease prevention. This payment method provides excellent control of healthcare costs under total prepayment and allows providers to take demand-side needs into account under capitation adequately [7].

Most of the previous studies in the evaluation of capitation payments have been based on cost studies of the entire population, and fewer studies have been conducted on a particular disease type alone. Therefore, it is of great significance and value to evaluate the impact of implementing capitation prepayment policy on patients with CHD in China. This study collected data related to medical care for residents in FN County and aimed to analyze the changes in medical expenses and health care utilization of CHD patients before and after the implementation of the capitation prepayment method.

## Methods

### Study design

In April 2015, the first batch of pilot towns in FN County adopted the “3 + 2 + 1” model, with three county hospitals taking the lead in building three medical communities. The first, second, and third medical community have formed Medical Community with three, two, and one central health centers and village clinics under their jurisdiction, respectively. With the gradual progress of the Medical Community pilots, on the basis of the first pilot model, the county further adopted the “7 + 5 + 4” model in May 2016 and “14 + 9 + 13” model in January 2017 as the second and third batches of Medical Community construction, expanding the scope of townships included in Medical Community until full township coverage was achieved.

Two townships were randomly selected in our study. JP Town, one of the first batch of pilot towns in 2015, was selected as the pilot group, and XT Town, which did not implement the capitation prepayment until early 2017, was selected as the control group. Patients diagnosed with CHD in two towns were defined as research subjects to compare and analyze medical expenses and health care utilization involved in the treatment of CHD in the two groups in 2014–2016, with the reform started in April 2015. The year 2014 was the pre-reform year, and the year 2015 and 2016 were the first two years of the post-reform period. In order to avoid the impact of different seasons on the prevalence of disease and to ensure year-to-year comparability, the annual data used in the analysis were from April to December of each year.

### Data sources

Data in 2014–2016 were collected from the Medical Insurance Expense Compensation Database of FN county. The data of CHD patients among contracted residents were used to analyze the changing trend of medical expenses and health service utilization resulting from the implementation of the reform.

The data information selected for this study included patient demographic information (age, gender, and poverty status), town code, medical institution level, diagnosis of disease, and medical expenses. Considering that patients’ comorbidity with other chronic diseases has a potential impact on medical expenses [8], we calculated the number of patients with other chronic diseases based on their diagnosis of disease.

The data were processed in this study using R4.0.4 software. Patients with missing personal characteristics and whose total costs exceeded the mean ± 3 times the standard deviation ($\bar \chi \pm 3s$) were excluded [9]. Finally, 2,138 outpatients were included in the pilot group (668 in 2014, 837 in 2015, and 633 in 2016), and 2,394 in the control group (758 in 2014, 838 in 2015, and 798 in 2016); 356 inpatients were included in the pilot group (100 in 2014, 131 in 2015, and 125 in 2016), and 539 in the control group (184 in 2014, 176 in 2015, and 179 in 2016).

### Outcomes

The measurement of the impact of capitation prepayment included both expense indicators and health service utilization indicators. Total medical expenses included health insurance payments and out-of-pocket costs. Outpatient costs mainly included the expense on drug, treatment, examination, laboratory, and other expenses. Inpatient costs primarily included the expense on drug, treatment, material, examination, laboratory, bed, nursing, and other expenses. Health service utilization indicators included visits to rural and county-level medical institutions, as well as visits within and outside the county.

### Statistical analysis

In recent years, there has been an increasing variety of policy evaluation methods, and common methods include synthetic control, segmented regression, propensity score matching (PSM), and difference-in-differences (DID) methods. Each of these methods has its own advantages and disadvantages, which are briefly described here [10]. Synthetic control method assesses the policy by comparing the test group with a synthetic control group, takes into account the combined information from multiple observations and points in time, and is able to provide more comprehensive and accurate results, but its validity is highly dependent on the chosen synthetic control group. Segmented regression is able to identify non-linear effects of policy on outcome variables and provide a comprehensive assessment of policy effects, but require a reasonable choice of segments to be made when using them, however this choice is often somewhat subjective. PSM is able to address the problem of sample selection bias by matching confounding variables in the balanced test and control groups, but does not avoid the problem of endogeneity. DID primarily assesses the effect produced by the policy by comparing the policy intervention group with the control group before and after the intervention [11]. This method can significantly address the endogeneity problem, but is prone to the sample selection bias problem. In this study, we use PSM-DID to match the pilot group and the control group to solve the sample selection bias problem by matching the covariates to maintain the parallel trend between the experimental group and the control group, and construct a DID model on this basis to reduce the influence of endogeneity problem, so as to obtain unbiased estimation of the effect of policy implementation.

PSM is a statistical method that can increase the comparability of trial and control groups under conditions of non-randomised treatment allocation, and it is one of the powerful tools currently available to address confounding bias in observational studies [12]. By calculating the propensity score value to combine all the variables and balance the difference between the trial and control groups at baseline, confounding factors are effectively controlled. By reviewing the literature and combining the actual variable indicators in the database, all the factors that may have an effect on the study indicators are treated as confounding variables and controlled by PSM. Propensity scores were generated using contract for the capitation prepayment as the dependent variable, and gender, age, number of comorbidities with other chronic diseases, and poverty status as independent variables. The caliper value matching method was adopted for 1:1 matching, and the caliper value was set to 0.2 [13]. After matching, if the absolute standardized mean difference (SMD) of all covariates between the two groups was less than 0.1, then the two groups could be considered to have reached equilibrium [14]. Ultimately, there were 3900 outpatients (1270 in 2014, 1476 in 2015, and 1154 in 2016) and 664 inpatients (188 in 2014, 232 in 2015, and 244 in 2016) after matching.

The progressive implementation of the capitation prepayment reform in FN County in 2015 resembles a “natural experiment” that meets the prerequisite of constructing a DID model. Based on PSM, this study further constructed a DID model for the two matched groups, and analyzed and compared the changes in medical expenses and health service utilization between the two groups during different time periods to obtain the “net effect” of the implementation of the capitation prepayment policy. The basic model of DID is as follows:

$$y{\text{ }} = {\beta _0} + {\beta _1}\left( {treat} \right) + {\beta _2}\left( {time} \right) + {\beta _3}\left( {treat*time} \right) + \varepsilon$$

In the above equation, ‘y’ indicates the outcome (each expense and the number of visits); ‘treat’ and ‘time’ are dummy variables; ‘treat’ indicates in implement of the capitation prepayment reform using 0 for the control group and 1 for the pilot group; ‘time’ indicates the time of policy implementation using 0 for before policy implementation and 1 for after policy implementation; ‘treat^*^time’ is the interaction term between policy and time; ‘β_0_’ is the intercept term; ‘β_3_’ is the estimated coefficient of the model measuring the impact of the policy; ‘ε’ is the residual term.

## Results

### Change in medical expenses between the two groups of patients from 2014 to 2016

**Fig. 1:**
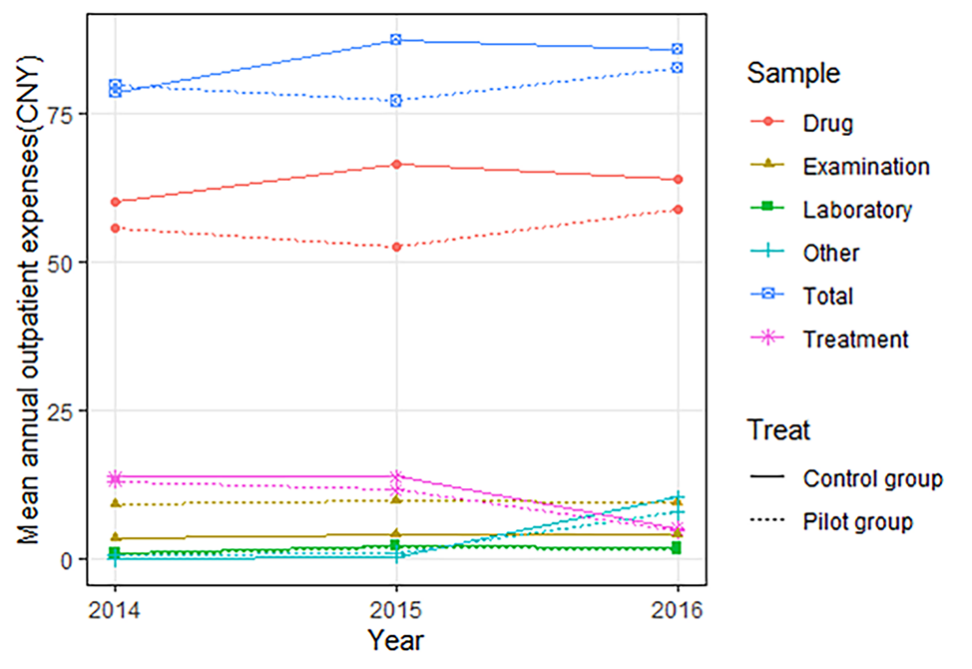
Annual expenses of CHD outpatients per capita (CNY), 2014–2016

The total annual expenses per capita and drug expenses of CHD outpatients presented the same trend from 2014 to 2016 in the pilot group, decreasing before 2015 and increasing since 2015 (Fig. 1), which were less than those in the control group. Both of the above expenses in the pilot group in 2016 were reduced by CNY 3.20 and CNY 4.93 compared to the control group, respectively. The annual treatment expenses per capita in the pilot and control groups descended after 2015 while annual other expenses per capita rose, both of which in the pilot group in 2016 were reduced by CNY 0.51 and CNY 2.53 compared to the control group, respectively. There was no significant change in the annual examination expenses per capita and annual laboratory expenses per capita between the two groups of outpatients in 2014–2016.

**Fig. 2:**
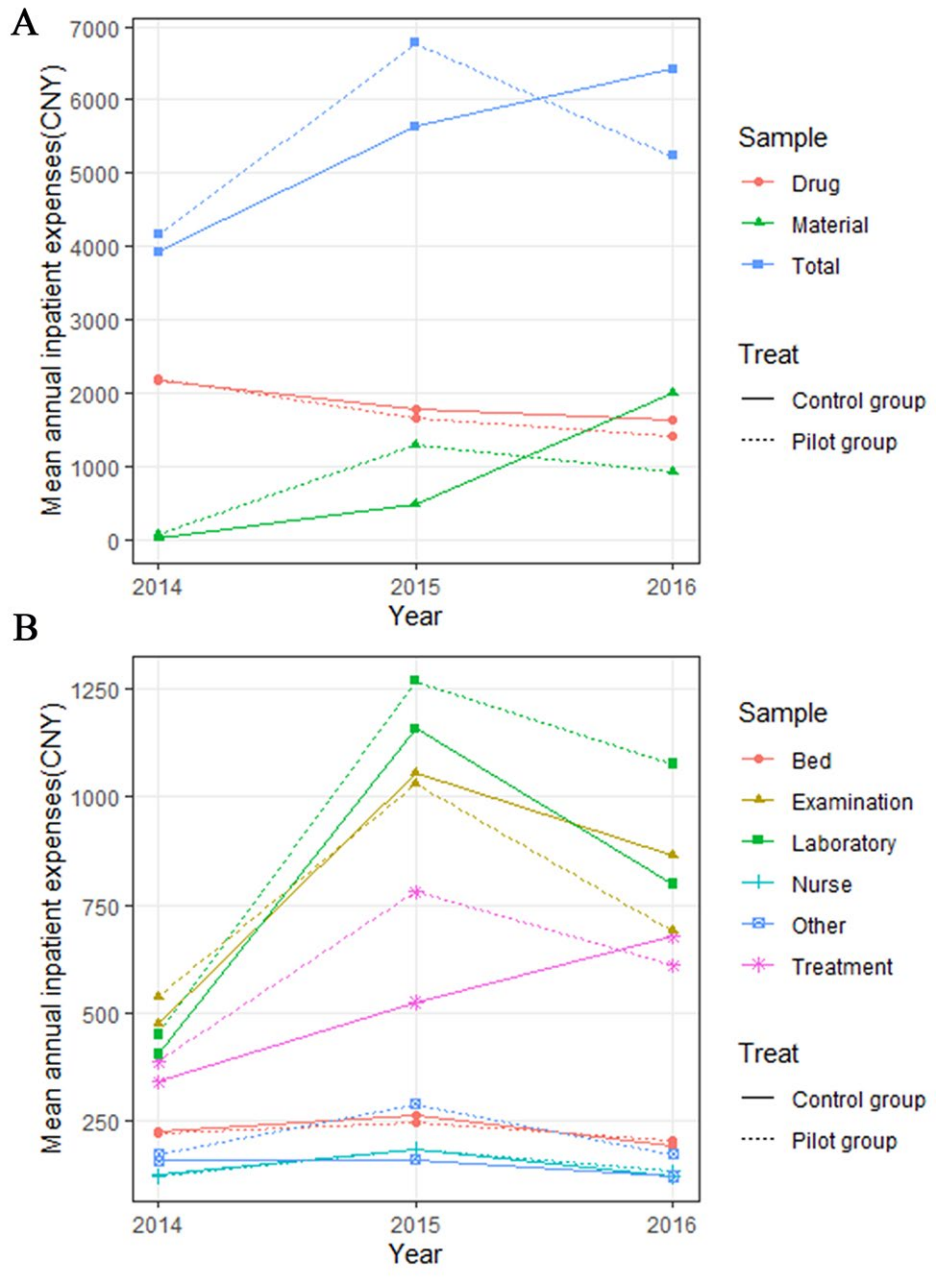
Annual expenses of CHD inpatients per capita (CNY), 2014–2016. Note: **(A)** Total annual expenses per capita, annual drug expenses per capita, and annual material expenses per capita of CHD inpatients (CNY); **(B)** Annual bed expenses per capita, annual examination expenses per capita, annual laboratory expenses per capita, annual nursing expenses per capita, annual treatment expenses per capita, and annual other expenses per capita of CHD inpatients (CNY).

The total annual expenses per capita and annual material expenses per capita of CHD inpatients increased before 2015 and decreased since 2015 in the pilot group (Fig. 2A), while the both have been continually rising in the control group. The total annual expenses per capita and annual material expenses per capita for the pilot group in 2016 were reduced by CNY 1127.24 and CNY 1075.60 compared to the control group, respectively. The annual drug expenses per capita for both the pilot and control groups presented a downward trend, with a greater decline in the pilot group. The annual drug expenses per capita for the pilot group was reduced by CNY 220.94 compared to the control group in 2016.

The annual laboratory expenses per capita and annual examination expenses per capita for both groups initially rose and then dropped after 2015 (Fig. 2B). Compared to the control group, the annual expenses per capita for laboratory tests was increased by CNY 277.33 and the annual expense per capita for examinations was decreased by CNY 173.06 for the pilot group in 2016. The annual treatment expenses per capita rose and then fell in the pilot group, while has been continually rising in the control group. The annual treatment expenses per capita for the pilot group was reduced by CNY 66.18 compared to the control group in 2016. There was little change in annual per capita bed expenses, nursing expenses and other expenses between the two groups.

### Health service utilization of CHD patients in two groups from 2014 to 2016

The number of outpatient visits in the pilot group decreased after the payment reform from 2.021 visits in 2014 to 1.809 visits in 2015, while it rose slightly in 2016, which all remained lower than the control group. The number of outpatient visits in the control group grew yearly from 1.917 to 2014 to 2.061 in 2016. Both groups showed a downward trend by year in the proportion of visits to rural medical institutions but an increasing trend in the proportion of visits to county health facilities (Table 1).

The number of patient hospitalizations decreased and then increased after the payment reform in the pilot group, while patient hospital days continued to decline after the reform to 3.040 days in 2016 which is far less than the hospital days of 7.637 days in the control group. The proportion of in-county visits in the pilot group improved from 80.95% to 87.44% after the reform, indicating inpatients gradually returned to the county. In contrast, in the control group, the number of hospitalizations declined by year while the number of hospital days has risen after a decline; the proportion of in-county visits decreased by year from 91.63% to 78.35%, while the proportion of out-county visits increased by year by 11.61% in 2016 compared to 2015 (Table 1).

**Table 1:** Health service utilization of CHD patients in two groups, 2014–2016

Year	Outpatient					Inpatient				
	n	Number of visits per year	Proportion of rural medical institutions visits(%)	Proportion of county medical institutions visits(%)		n	Number of hospitalizations per year	Number of hospital days per year	Proportion of within county visits(%)	Proportion of outside county visits(%)
2014										
Pilot group	668	2.021	99.48	0.52		100	1.680	4.610	80.95	19.05
Control group	758	1.917	99.24	0.76		184	1.429	5.886	91.63	8.37
2015										
Pilot group	837	1.809	99.27	0.73		131	1.557	4.344	84.31	15.69
Control group	838	1.933	99.07	0.93		176	1.358	5.358	89.96	10.04
2016										
Pilot group	633	1.855	97.62	2.38		125	1.784	3.040	87.44	12.56
Control group	798	2.061	98.91	1.09		179	1.291	7.637	78.35	21.65

### Characteristics of CHD patients between the two groups before and after using PSM

**Fig. 3:**
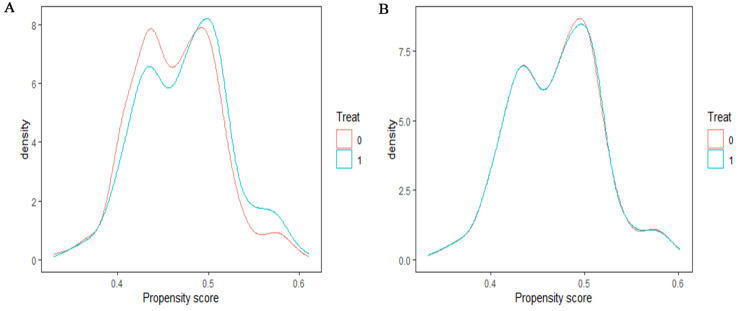
Distribution of propensity score for outpatients in both groups in 2014. Note: **(A)** Distribution of propensity score for outpatients before PSM in 2014; **(B)** Distribution of propensity score for outpatients after PSM in 2014

**Fig. 4:**
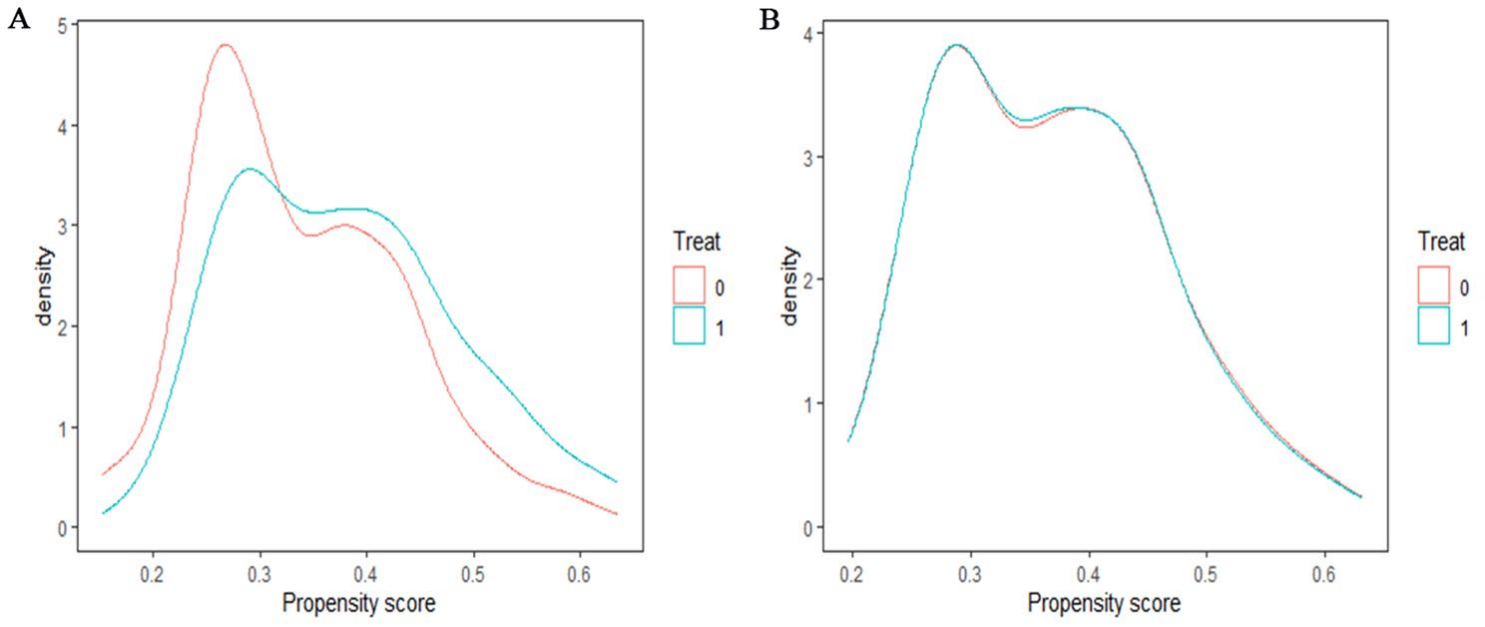
Distribution of propensity score for inpatients in both groups in 2014. Note: **(A)** Distribution of propensity score for inpatients before PSM in 2014; **(B)** Distribution of propensity score for inpatients after PSM in 2014

The distributions of personal characteristics before and after using PSM for outpatients and inpatients with CHD in pilot and control groups were presented in Tables 2 and 3. Overall, the majority of CHD patients were female, elderly, with a combination of ≤ 10 other chronic diseases, and non-poor, and inpatients were older than outpatients. All SMDs were less than 0.1 in both groups after adjusted by PSM, indicating a balanced distribution of patients’ personal characteristics in the pilot and control groups. The propensity score curves of the two outpatient groups in 2014 after PSM almost overlapped, indicating a better matching effect (Fig. 3), as well as those of inpatient groups (Fig. 4). The data used in subsequent parts of this study are originated from patients after PSM.

**Table 2:** Comparison of pre and post PSM characteristics of outpatients in 2014–2016

Year	Variables	Before using PSM			After using PSM		
		Pilot group	Control group	*SMD*	Pilot group	Control group	*SMD*
2014(n)		668	758		635	635	
	Sex, n (%)						
	Male	290 (43.4)	315 (41.6)	0.038	275 (43.3)	281 (44.3)	0.019
	Female	378 (56.6)	443 (58.4)		360 (56.7)	354 (55.7)	
	Age ($\bar \chi \pm s$*± s*)	60.34 ± 15.36	60.74 ± 14.81	0.027	60.23 ± 15.51	60.76 ± 14.44	0.035
	Number of comorbidities, n (%)						
	0	26 (3.9)	32 (4.2)	0.174	26 (4.1)	23 (3.6)	0.005
	1–5	300 (44.9)	396 (52.2)		300 (47.2)	303 (47.7)	
	6–10	288 (43.1)	297 (39.2)		275 (43.3)	276 (43.5)	
	> 10	54 (8.1)	33 (4.4)		34 (5.4)	33 (5.2)	
	Poverty Status, n (%)						
	Poor	162 (24.3)	186 (24.5)	0.007	155 (24.4)	154 (24.3)	0.004
	Non-poor	506 (75.7)	572 (75.5)		480 (75.6)	481 (75.7)	
2015(n)		837	838		738	738	
	Sex, n (%)						
	Male	368 (44.0)	330 (39.4)	0.093	308 (41.7)	307 (41.6)	0.003
	Female	469 (56.0)	508 (60.6)		430 (58.3)	431 (58.4)	
	Age ($\bar \chi \pm s$*± s*)	60.18 ± 16.05	62.07 ± 14.97	0.121	61.19 ± 15.59	61.11 ± 14.96	0.005
	Number of comorbidities, n (%)						
	0	37 (4.4)	38 (4.5)	0.227	37 (5.0)	22 (3.0)	0.006
	1–5	346 (41.3)	436 (52.0)		333 (45.1)	364 (49.3)	
	6–10	370 (44.2)	321 (38.3)		323 (43.8)	309 (41.9)	
	> 10	84 (10.1)	43 (5.2)		45 (6.1)	43 (5.8)	
	Poverty Status, n (%)						
	Poor	207 (24.7)	238 (28.4)	0.083	195 (26.4)	195 (26.4)	<0.001
	Non-poor	630 (75.3)	600 (71.6)		543 (73.6)	543 (73.6)	
2016(n)		633	798		577	577	
	Sex, n (%)						
	Male	269 (42.5)	329 (41.2)	0.026	250 (43.3)	253 (43.8)	0.010
	Female	364 (57.5)	469 (58.8)		327 (56.7)	324 (56.2)	
	Age ($\bar \chi \pm s$*± s*)	62.20 ± 14.59	62.91 ± 14.78	0.048	62.06 ± 14.82	62.31 ± 14.73	0.017
	Number of comorbidities, n (%)						
	0	21 (3.3)	44 (5.5)	0.332	20 (3.5)	18 (3.1)	0.008
	1–5	244 (38.5)	391 (49.0)		244 (42.3)	248 (43.0)	
	6–10	289 (45.7)	330 (41.4)		277 (48.0)	278 (48.2)	
	> 10	79 (12.5)	33 (4.1)		36 (6.2)	33 (5.7)	
	Poverty status, n (%)						
	Poor	178 (28.1)	270 (33.8)	0.124	172 (29.8)	166 (28.8)	0.023
	Non-poor	455 (71.9)	528 (66.2)		405 (70.2)	411 (71.2)	

Note: SMD, standardized mean difference

**Table 3:** Comparison of pre and post PSM characteristics of inpatients from 2014–2016

Variables		Before using PSM			After using PSM			
		Pilot group	Control group	*SMD*	Pilot group	Control group	*SMD*	
2014(n)		100	184		94	94		
	Sex, n (%)							
	Male	38 (38.0)	61 (33.2)	0.101	35 (37.2)	39 (41.5)	0.087	
	Female	62 (62.0)	123 (66.8)		59 (62.8)	55 (58.5)		
	Age ($\bar \chi \pm s$*± s*)	67.32 ± 11.26	66.89 ± 11.23	0.038	67.17 ± 11.39	66.14 ± 10.74	0.093	
	Number of comorbidities, n (%)							
	0	2 (2.0)	11 (6.0)	0.410	2 (2.1)	2 (2.1)	<0.001	
	1–5	43 (43.0)	101 (54.9)		43 (45.7)	43 (45.7)		
	6–10	40 (40.0)	62 (33.7)		40 (42.6)	40 (42.6)		
	> 10	15 (15.0)	10 (5.4)		9 (9.6)	9 (9.6)		
	Poverty status, n (%)							
	Poor	38 (38.0)	59 (32.1)	0.124	36 (38.3)	33 (35.1)	0.066	
	Non-poor	62 (62.0)	125 (67.9)		58 (61.7)	61 (64.9)		
2015(n)		131	176		116	116		
	Sex, n (%)							
	Male	46 (35.1)	66 (37.5)	0.050	41 (35.3)	41 (35.3)	<0.001	
	Female	85 (64.9)	110 (62.5)		75 (64.7)	75 (64.7)		
	Age ($\bar \chi \pm s$*± s*)	67.79 ± 11.68	69.26 ± 11.63	0.126	69.00 ± 11.56	68.46 ± 12.00	0.046	
	Number of comorbidities, n (%)							
	0	6 (4.6)	8 (4.5)	0.337	4 (3.4)	2 (1.7)	0.026	
	1–5	42 (32.1)	88 (50.0)		42 (36.2)	47 (40.5)		
	6–10	69 (52.6)	71 (40.4)		61 (52.6)	59 (50.9)		
	> 10	14 (10.7)	9 (5.1)		9 (7.8)	8 (6.9)		
	Poverty status, n (%)							
	Poor	43 (32.8)	59 (33.5)	0.015	40 (34.5)	42 (36.2)	0.036	
	Non-poor	88 (67.2)	117 (66.5)		76 (65.5)	74 (63.8)		
2016(n)		125	179		122	122		
	Sex, n (%)							
	Male	48 (38.4)	85 (47.5)	0.184	48 (39.3)	45 (36.9)	0.051	
	Female	77 (61.6)	94 (52.5)		74 (60.7)	77 (63.1)		
	Age ($\bar \chi \pm s$*± s*)	66.25 ± 11.91	67.72 ± 11.17	0.127	66.45 ± 11.89	66.87 ± 11.40	0.036	
	Number of comorbidities, n (%)							
	0	5 (4.0)	6 (3.3)	0.128	5 (4.1)	4 (3.3)	0.057	
	1–5	51 (40.8)	81 (45.3)		50 (41.0)	52 (42.6)		
	6–10	54 (43.2)	81 (45.3)		52 (42.6)	56 (45.9)		
	> 10	15 (12.0)	11 (6.1)		15 (12.3)	10 (8.2)		
	Poverty status, n (%)							
	Poor	41 (32.8)	75 (41.9)	0.188	39 (32.0)	37 (30.3)	0.035	
	Non-poor	84 (67.2)	104 (58.1)		83 (68.0)	85 (69.7)		

Note: SMD, standardized mean difference

### Changes in medical expenses for CHD patients per capita from 2014 to 2016

Two years after the implementation of the capitation prepayment reform, the annual outpatient medical expenses per capita and annual growth rate in the pilot group were lower than those in the control group. Annual inpatient medical expenses per capita and annual growth rate in the pilot group were higher than those in the control group in 2015, while lower than those in the control group in 2016 (Table 4).

**Table 4:** Changes in annual medical expenses for CHD patients per capita in 2014–2016

Year	Outpatient				Inpatient			
	Pilot group		Control group		Pilot group		Control group	
	Capita cost (CNY)	Growth rate (%)	Capita cost (CNY)	Growth rate (%)	Capita cost (CNY)	Growth rate (%)	Capita cost (CNY)	Growth rate (%)
2014	79.61	-	75.71	-	4954.65	-	4271.07	-
2015	76.79	-3.54	86.84	14.70	6776.54	37.77	5592.27	30.93
2016	83.64	5.06	86.02	13.62	5410.85	9.21	6637.11	55.40

### DID analysis of medical expenses and health service utilization for CHD outpatients

The DID model in Table 5 showed that in the first year of implementing the capitation prepayment reform, compared to the control group, total outpatient expenses reduced by CNY 13.953 and drug expenses decreased by CNY 11.289 in the pilot group; the Medicare payment in the pilot group declined by CNY 8.707 and out-of-pocket expenses did not change significantly; there was a decreasing trend in treatment expenses, examination expenses and laboratory expenses in the pilot group, but the difference was not statistically significant. Regarding the health service utilization of outpatients, there was no remarkable change in the number of visits to rural medical institutions and county medical institutions in the pilot group compared with the control group in 2015.

In the second year of implementation of the capitation prepayment reform, compared with the control group, there was no significant change in total outpatient expenses, drug expenses, treatment expenses, examination expenses and laboratory expenses, while other expenses decreased by CNY 3.123 in the pilot group; the pilot group had a CNY 6.841 reduction in the Medicare payment, but out-of-pocket expenses did not change significantly. In terms of health service utilization of outpatients, the number of visits to rural medical institutions reduced by 0.403 in the pilot group compared to the control group in 2016 (Table 5).

**Table 5:** DID model of outpatient expenses and health service utilization in both groups

Variables	n	Pilot group	Control group	Time	Treat	Time^*^treat
Total expenses (CNY)						
2014	635	79.61	75.71	-	-	-
2015	738	76.79	86.84	11.132^**^	3.899	-13.953^**^
2016	577	83.64	86.02	10.313^**^	3.899	-6.278
Medicare payment expenses (CNY)						
2014	635	41.81	39.67	-	-	-
2015	738	40.26	46.83	7.163^***^	2.138	-8.707^**^
2016	577	45.47	50.18	10.506^***^	2.138	-6.841^*^
Out-of-pocket expenses (CNY)						
2014	635	37.80	36.04	-	-	-
2015	738	36.52	40.01	3.969	1.761	-5.246
2016	577	38.17	35.85	-0.193	1.761	0.562
Drug expenses (CNY)						
2014	635	55.76	58.18	-	-	-
2015	738	52.68	66.39	8.209^**^	-2.424	-11.289^**^
2016	577	59.41	64.04	5.855	-2.424	-2.201
Treatment expenses (CNY)						
2014	635	13.12	13.51	-	-	-
2015	738	11.76	13.77	0.262	-0.383	-1.627
2016	577	4.76	5.26	-8.250^***^	-0.383	-0.110
Examination expenses (CNY)						
2014	635	9.19	2.70	-	-	-
2015	738	9.60	3.93	1.232	6.486^***^	-0.820
2016	577	9.99	4.10	1.393	6.486^***^	-0.595
Laboratory expenses (CNY)						
2014	635	0.92	1.27	-	-	-
2015	738	1.84	2.48	1.209	-0.343	-0.291
2016	577	1.67	2.12	0.857	-0.343	-0.109
Other expenses (CNY)						
2014	635	0.61	0.05	-	-	-
2015	738	0.89	0.27	0.220	0.560	0.059
2016	577	7.81	10.37	10.319^***^	0.560	-3.123^***^
Visits to rural medical institutions						
2014	635	1.992	1.849	-	-	-
2015	738	1.799	1.894	0.045	0.143	-0.238
2016	577	1.797	2.057	0.208^*^	0.143	-0.403^**^
Visits to county medical institutions						
2014	635	0.011	0.013	-	-	-
2015	738	0.014	0.020	0.008	-0.002	-0.005
2016	577	0.045	0.023	0.010	-0.002	0.024

Note: * indicates *P* < 0.05, ** indicates *P* < 0.01, *** indicates *P* < 0.001

### DID analysis of medical expenses and health service utilization for CHD inpatients

The DID model in Table 6 showed that in the first year of implementing the capitation prepayment reform, there were no statistically significant changes in the total hospitalization expenses, drug expenses, treatment expenses, material expenses and examination expenses in the pilot group compared to the control group. In respect of health service utilization of inpatients, compared with the control group, there was no statistically significant change in the number of visits to rural medical institutions and county medical institutions in the pilot group, nor was there a significant change in the number of visits within and outside the county.

In the second year of implementation of the capitation prepayment reform, there was no statistically significant change in medical expenses in the pilot group compared to the control group (Table 6). In regard to health service utilization of inpatients, compared with the control group, the number of visits to rural medical institutions rose by 0.829 in the pilot group; the number of visits to county medical institutions was not significantly different; the number of visits within the county increased by 0.750 and the number of visits outside the county decreased by 0.222 (Table 6).

**Table 6:** DID model of inpatient expenses and health service utilization in both groups

Variables	n	Pilot group	Control group	time	treat	time^*^treat
Total inpatient expenses (CNY)						
2014	94	4954.65	4271.07	-	-	-
2015	116	6776.54	5592.27	1321.200	683.600	500.700
2016	122	5410.85	6637.11	2366.000^*^	683.600	-1909.800
Medicare payment expenses (CNY)						
2014	94	2905.82	2900.41	-	-	-
2015	116	4225.51	3785.54	885.137	5.414	434.548
2016	122	3579.73	3911.05	1010.638	5.414	-336.730
Out-of-pocket expenses (CNY)						
2014	94	2048.83	1366.56	-	-	-
2015	116	2641.45	1936.06	569.490	682.260	23.130
2016	122	2111.64	2863.85	1497.300^**^	682.260	-1434.500
Drug expenses (CNY)						
2014	94	2205.17	2227.24	-	-	-
2015	116	1707.83	1770.81	-456.420	-22.070	-40.920
2016	122	1429.14	1571.55	-655.680^*^	-22.070	-120.350
Treatment expenses (CNY)						
2014	94	438.64	400.44	-	-	-
2015	116	763.06	519.54	119.100	38.190	205.330
2016	122	609.70	682.06	281.620	38.190	-110.560
Material expense (CNY)						
2014	94	136.21	31.68	-	-	-
2015	116	1304.27	500.36	468.680	104.530	699.380
2016	122	941.92	2203.84	2172.160^***^	104.530	-1366.440
Examination expenses (CNY)						
2014	94	633.94	503.24	-	-	-
2015	116	1053.60	1004.20	500.960^***^	130.700	-81.300
2016	122	697.36	918.84	415.600^**^	130.700	-352.200
Laboratory expenses (CNY)						
2014	94	514.91	426.15	-	-	-
2015	116	1287.93	1142.17	716.020^***^	88.760	57.000
2016	122	1084.77	775.28	349.120^**^	88.760	220.730
Bedding expenses (CNY)						
2014	94	222.10	241.22	-	-	-
2015	116	252.05	265.22	24.002	-19.117	5.949
2016	122	206.02	183.41	-57.810	-19.117	41.730
Nursing expense (CNY)						
2014	94	122.61	129.81	-	-	-
2015	116	190.96	189.10	59.287	-7.198	9.055
2016	122	135.44	107.59	-22.220	-7.198	35.044
Other expenses (CNY)						
2014	94	196.51	174.95	-	-	-
2015	116	159.40	141.18	-33.770	21.568	-3.339
2016	122	177.17	109.72	-65.230	21.568	45.890
Visits to rural medical institutions						
2014	94	0.574	0.936	-	-	-
2015	116	0.526	0.500	-0.436^*^	-0.362	0.388
2016	122	0.934	0.467	-0.469^*^	-0.362	0.829^*^
Visits to county medical institutions						
2014	94	0.638	0.543	-	-	-
2015	116	0.681	0.759	0.216	0.096	-0.173
2016	122	0.631	0.615	0.072	0.096	-0.079
Visits within the county						
2014	94	1.213	1.479	-	-	-
2015	116	1.207	1.259	-0.220	-0.266	0.214
2016	122	1.566	1.082	-0.397	-0.266	0.750^*^
Visits outside the county						
2014	94	0.287	0.106	-	-	-
2015	116	0.241	0.164	0.057	0.181^*^	-0.103
2016	122	0.221	0.262	0.156^*^	0.181^*^	-0.222^*^

Note: * indicates *P* < 0.05, ** indicates *P* < 0.01, *** indicates *P* < 0.001

## Discussion

Existing research on payment policy reform has shown that capitation can optimize patient clinical outcomes and reduce medical costs [15–19], thus payment reform is urgently needed for China to achieve its health care reform goals. However, there are currently fewer studies focusing on specific diseases and they are mainly concentrated on diabetes. We used patient-level data and PSM-DID model to examine the impact of payment method changes on medical expenses and health service utilization for patients with CHD based on a community policy intervention program in a county in China. Our study found that capitation prepayment effectively enhanced the service capacity of medical institutions, constrained rising expenses of CHD outpatients, reduced patients’ burden of disease, and improved in-county visit rates. The findings will provide further policy support or evidence for medical insurance payment reform in developing countries.

### Capitation prepayment approach effectively promoted integrated health care service delivery

The construction of the Medical Community usually focuses on improving the service capacity of primary healthcare institutions, however, if the total payment method is implemented for individual medical institutions within a medical community, there will be a competition for services among medical institutions and it is extremely difficult to form an integrated medical and health service provision [20]. FN County implements a capitation prepayment method for Medical Community, which allocates medical insurance funds according to the number of participants covered by Medical Community and the leading hospital is responsible for the use of the funds; in principle, overspending is not compensated and the balance is distributed according to the ratio of the county hospital to the township health center to the village health office of 6:3:1. This packaged payment approach associates the interests of tiered institutions within Medical Community and significantly improves the competition among them. In addition, the county’s basic public health funds are also assigned to Medical Community in accordance with the resident population under its jurisdiction, which is distributed by its lead hospitals according to the quantity and quality of basic public health services provided by the township health center.

The packaged payment to Medical Community effectively mobilizes the intrinsic motivation of institutions at all levels within Medical Community to provide appropriate services. County hospitals proactively established Medical Clusters with extra-territorial specialty hospitals, targeted strengthening the specialty service capacity by expanding digitally integrated operating rooms, equipping new medical equipment, developing tele-medicine systems, building academician workshops, inviting (experts) in and sending (doctors) out, etc. to accelerate the training of discipline leaders with specialist backbone physicians, explore the establishment of multidisciplinary joint diagnosis modalities and enhance the medical service capacity of medical personnel, so that county hospitals’ “treating serious diseases” level has been significantly improved [21]. Meanwhile, the county hospital trusted township health centers by sending the director of the health center, co-building departments, helping with consultation, free training, and other ways to enhance the ability of “treating common diseases” for the township health center. In addition, township health centers and county hospitals have implemented the “50 + N” and “100 + N” disease diagnosis and treatment catalogues respectively, i.e., there are 50 diseases that must be treated in the township health centers, and N is the number of diseases that can be appropriately added to the 50 diseases depending on the service capacity of the township health centers. Similarly, 100 diseases must be treated in county hospitals and cannot be transferred out of the county, the health-seeking behavior of residents is relatively standardized and consistent. The proportion of CHD patients hospitalized in rural medical institutions in FN County reached 52.29% in 2016, increasing by 13.99% compared with the pre-reform period, which effectively relieved the pressure of receiving medical treatment in county medical institutions.

A study from Ontario in Canada proved that patients in the capitation model had lower morbidity compared with the fee-for-service model [22]. Under the principle of surplus for yourself, medical institutions at all levels within the Medical Community actively launched preventive services and reinforced the contracted services of family doctors in order to make residents in the county less sick, later sick, and minor sick [23]. FN County People’s Hospital took the lead in setting up a chronic disease management team at the village level, which composes of doctors at the county, township, and village levels, to promote appropriate technology in village health offices and comprehensively carry out health management services for the elderly over 65 years old and patients with hypertension, diabetes, CHD as well as stroke as key management groups, make the reduction of morbidity rate of residents in the district as a key indicator of performance assessment to build a solid “prevention before diseases” system. Medical institutions within the Medical Community provide continuous medical and health services with complex functions and positionings, for facilitating the realization of the health goal of “treating serious illnesses within the county, seeing common illnesses nearby, and preventing before disease”.

### Capitation prepayment effectively constrained rising expenses of CHD outpatients

The DID model showed that in the first year of implementing the capitation prepayment reform in FN County, compared to the control group, total outpatient expenses reduced by CNY 13.953, drug expenses decreased by CNY 11.289, and Medicare payments declined by CNY 8.707 in the pilot group. Our findings suggested the capitation prepayment method has efficiently controlled the excessive rise in CHD outpatient expenses, enhanced the efficiency of health insurance fund utilization, and improved the situation that expenditure over income in the past. Nguyen et al. found that the implementation of capitation payment reform resulted in lower per capita medical expenditure and per capita drug expenditure in hospitals, which is consistent with the results of this study [24]. FN County has seen a continuous decline in Medicare payments for CHD outpatients and insignificant changes in out-of-pocket costs during the two years after the implementation of the payment reform, indicating that providers are paying attention to the use of Medicare funds. It might ameliorate the issue of “moral hazard” arising from information asymmetry between medical insurance providers and medical institutions. In addition, during the two years of implementation of the capitation prepayment reform, there was no statistically significant change in medical expenses in the pilot group for inpatients compared to the control group. FN County implemented the capitation prepayment method within Medical Community while inpatients medical expenses for some types of illnesses are applied for payment by disease, including CHD, which may lead to the insignificant effect of the reform on inpatient expenses of CHD patients in the DID model.

Under the capitation prepayment system, the interests of physicians and hospitals as providers of healthcare are often aligned. The medical insurance fund prepaid to the hospital is known and the difference between the actual medical expenses and the prepaid amount has a direct impact on the provider due to the “hypothesis of rational man”, with the biggest benefit to the provider when the difference is the largest [25]. After the implementation of the capitation prepayment in the pilot group in the sample area, the percentage of the use of Shenmai Injection has decreased significantly compared to that of the control group (the percentage of the use of Shenmai Injection in the pilot group in 2015 was 6.01% compared with 11.27% in the control group) and an increase in the use of Compound Danshen Tablets. It is worth noting that both medicines are effective drugs for treating CHD, which can both replenish Qi and activate blood, but Shenmai Injection is more expensive. It can be seen that physicians as immediate decision makers in medical practice, prepayment system creates certain incentives for physicians [26]. Doctors engage in autonomous fee-control and attempt to reduce patients’ medical expenses by lessening unnecessary medical treatment items, including stopping some patients from using medical insurance reimbursement to ask for more drugs, and guiding patients to use medical resources wisely. The capitation prepayment system enables the sharing of benefits and risks among medical institutions within the Medical Community, thus increasing the initiative of medical institutions at all levels to save health insurance funds [27], encouraging hospitals to strengthen internal management and reinforce the regulation of drug-to-medical ratio together with antibiotic use indicators to standardize doctors’ medical behavior and control inappropriate drug profits. However, for the purpose of reducing the risk and cost of treatment, medical institutions may choose patients with milder conditions or better health, reduce the use of high-end technologies, shirk or refuse patients with critical illnesses [28]. In addition, in order to control medical costs, medical service providers may lower the standard of treatment, reduce the number of health services, or even provide fewer or insufficient essential medical services, so that the quality of medical services cannot be guaranteed in the course of medical services [29]. Therefore, constructing a hybrid payment method, increasing supervision and strengthening education on medical ethics are particularly crucial to ensuring the quality of medical services.

### Capitation prepayment improved in-county visit rates for CHD

The DID results showed that the number of visits for inpatients in the pilot group within the county increased by 0.750 and the number of visits outside the county decreased by 0.222, suggesting that more CHD patients chose to stay in the county for inpatient care, consistent with the findings of Yu et al. [30]. The reasons for in-county reflux in patients include three aspects. Firstly, in-county consultation can save the patient’s household transportation and accommodation costs and reduce the burden of the patient’s expenses. Meanwhile, after the service capacity of medical institutions in the county was enhanced, patients’ medical service demand can be met in the county and patients proactively returned to the county. Secondly, the establishment and improvement of the medical insurance fund compensation management system for referral at each level has standardized patients’ medical behavior, gradually returning medical services to orderly treatment. Thirdly, the medical insurance compensation scheme implemented differential reimbursement policies for medical expenses at different levels of medical institutions, allowing the price leverage of medical insurance to be applied and objectively guiding patients to rational medical behavior.

### Suggestions

Based on the results of this study, we propose the following recommendations. Firstly, as the process of aging in China deepens, there is an irreversible tendency for patients, particularly those with chronic diseases, to prolong and exacerbate their illnesses, and medical technology is constantly advancing, which will drive up the cost of patient care over time. Therefore, when assessing medical institutions, health administrative departments should distinguish variations among diseases to assess expenses and avoid affecting patients’ health rights as a result. Secondly, the capitation prepayment has a determining influence on the reform process of the tiered medical system. Although there was a lack of tangible evidence of the effectiveness of the capitation prepayment reform during the study period, the role of the capitation prepayment in promoting prevention and facilitating the shift of the health care from a treatment-centered to a patient-health-centered approach cannot be dismissed. In the context of the implementation of DRG or DIP reform for inpatient medical expenses in China, it is recommended that the exploration of the implementation of capitation prepayment reform for outpatient medical expenses should still be expanded. Thirdly, while focusing on controlling medical expenses, it is imperative to curb supply-side moral hazards. The implementation of the pre-payment system needs to refine the supervision of service delivery in medical institutions and monitor the number of consultations, rejections, and various expenses in real-time [31], so as to guide medical institutions and doctors to rationalize treatment.

### Limitations

While the capitation prepayment is effective in controlling cost escalation, there are some potentials for providers to over-emphasize medical expenses for the same treatment outcome which may lower the quality of medical services [22]. Due to the absence of data on clinical indicators related to health outcomes of CHD patients in this study, it is not yet possible to measure and compare the quality of services provided by healthcare providers, nevertheless, this is not the focus of this study.

FN County implemented the capitation prepayment reform as a pilot in April 2015 in some townships and achieved comprehensive coverage within the county in January 2017, hence only two years of data were available for the control groups in this study. Moreover, the effect of the reform may have a lag, resulting in an inadequate demonstration of the effect of implementing the capitation prepayment reform, therefore the long-term impact of this payment method is still warranted.

## Conclusions

This study provides new evidence on the impact of using the capitation prepayment on medical expenses and health service utilization for CHD patients in China. The capitation prepayment can reduce patients’ burden of disease, guide patients’ orderly medical behavior and regulate doctors’ service delivery, promote the provision of preventive services and facilitate the transformation of health care services from treatment-oriented to patient health-oriented.

## Data Availability

Since our team signed a confidentiality agreement in this project, we cannot disclose the raw data. The datasets generated and/or analysed during the current study are not publicly available but are available from the corresponding author on reasonable request.
